# Homeless in Scotland: An Oral Health and Psychosocial Needs Assessment

**DOI:** 10.3390/dj6040067

**Published:** 2018-12-01

**Authors:** Laura Beaton, Emma Coles, Ruth Freeman

**Affiliations:** 1 Dental Health Services Research Unit, University of Dundee, Dundee DD1 4HN, Scotland, UK; r.e.freeman@dundee.ac.uk; 2 Nursing, Midwifery and Allied Health Professions Research Unit, University of Stirling, Stirling FK9 4NF, Scotland, UK; emma.coles@stir.ac.uk

**Keywords:** homeless persons, oral health, delivery of health care, dental health services

## Abstract

The aim of this research was to conduct an oral health and psychosocial needs assessment of a homeless population in Scotland to determine the levels of unmet need and provide recommendations for oral health improvement. A non-probability convenience sample of homeless people residing in seven Scottish Health Boards was collected. All consenting participants were asked to complete a questionnaire assessing their health and psychosocial needs, dental anxiety, and oral health-related quality of life. The participants’ oral health was examined by a trained and calibrated dentist and dental nurse. Eight hundred and fifty-three homeless people consented to take part. Participants had a mean D_3cv_MFT score of 16.9 (95% CI: 16.3, 17.6). Dental anxiety was high, with 20% scoring as dentally phobic. Respondents with higher dental anxiety were found to have significantly greater mean numbers of filled teeth than those with lower dental anxiety (*t* = −2.9, *p* < 0.05). Common oral health impacts were painful aching and discomfort while eating, experienced occasionally by 31% and 27% of the respondents, respectively. Fifty-eight percent of participants were found to have a depressive illness, and obvious decay experience was significantly higher among this section of participants (*t* = −4.3, *p* < 0.05). Homeless people in Scotland were found to be in need of a more accessible dental service than is currently available. An enhanced service should meet the oral health and psychosocial needs of this population to improve their oral health and quality of life.

## 1. Introduction

In Scotland, between 2012 and 2013, 39,827 homelessness applications were made. Sixty-five percent of those making the applications were single people. The majority of applications (55%) were made by men. Thirty percent of homeless applications were from single households with children (i.e., one parent families). These were predominantly women (74%). While this, overall, represented a fall by some 13% in homelessness applications, the proportion of those considered as a priority, or frontline homeless, had risen by 5% between 2011 and 2013. This suggested that the number of those with an acute housing need had not fallen, but rather had increased [1]. While these statistics represent official homelessness figures, the true number of people experiencing homelessness in Scotland remains unknown, due to the concept of “hidden homelessness” and the inherent difficulties when defining homelessness. Therefore, the definition of homelessness used here was the European Typology of Homelessness, which defines homelessness in terms of accommodation [2]. Therefore, those who are roofless and those who are houseless (residing in insecure and/or inadequate accommodation) are characterized as experiencing homelessness.

Previous research has established that people experiencing homelessness have poor general and oral health. Hwang found that people experiencing homelessness had poor general health, a “high burden of illness” and “a greatly increased risk of death” [3] (pp. 232, 230). Regarding oral health, Daly et al. found that the oral health of people experiencing homelessness was poor, with a great need for restorative, oral hygiene, and periodontal treatment [4]. Figueiredo et al. confirmed that homeless populations had poor oral health, poor attendance, a reliance on emergency treatment, and unmet treatment needs [5]. 

The healthcare needs of homeless people in Scotland have long been recognised by the Scottish Government. In 2005 they produced the Health and Homelessness Standards, to ensure that National Health Service (NHS) Boards gave special consideration to improving the understanding, planning, and treatment of homeless people within their Board areas [6]. This was extended to the Action Plan for Improving Oral Health and Modernizing NHS Dental Services in Scotland (Dental Action Plan) in 2005. The Dental Action Plan recognised homeless people as a priority group, requiring tailored oral health care [7]. By 2012, the Scottish Government perceived that homeless people represented ‘adults most in need’, and in their Priority Group Strategy of 2012 [8] called for accessible oral health care facilities:

‘*Homeless people have a variety of challenges facing them. Many are affected by poor general health, low self-esteem and poorer than average dental health. They may have problems accessing facilities to carry out oral self-care and often have difficulty in accessing dental services.*’

(p. 2)

With the emphasis on accessible health care and preventive programs, the need to understand the oral health status together with homeless people’s experiences of dental health care was seen as a first step in developing accessible services [9]. Therefore, the aim of this survey was to assess the oral health and psychosocial needs of homeless people across Scotland to allow recommendations for accessible dental health services to be made and to inform future oral health policy.

## 2. Materials and Methods

### 2.1. The Sample

**Figure 1:**
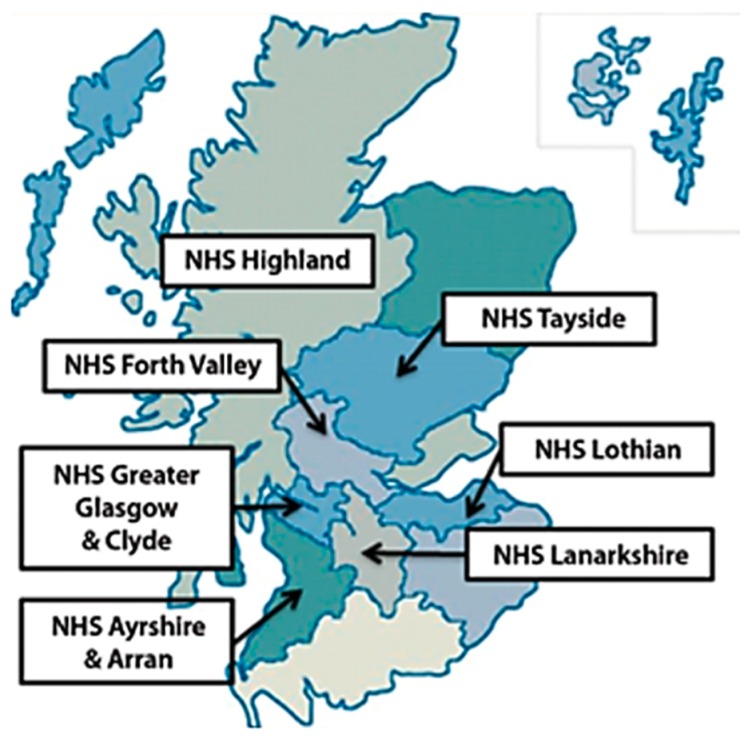
NHS Boards that participated in the Smile4life needs assessment (image reproduced from the Smile4life Report [12].

A non-probability convenience sample of homeless people residing in seven National Health Services (NHS) Boards across Scotland was collected. In Scotland there are 14 NHS Boards, each representing a different geographical region, which provide primary and secondary level health care services to the population. In Scotland and in the United Kingdom, the NHS meets the needs of the population; is based on clinical need, not a person’s ability to pay for treatment; and, it provides treatment that is free at the point of delivery [10]. The participating Scottish NHS Boards represented a mix of urban and rural localities (Figure 1). 

Non-probability convenience sampling was used due to the transient nature of those experiencing homelessness, which can make them a difficult population to reach [11]. A number of different localities in each NHS Board were visited several times, in order to generate a snowball effect and thus maximize the number of participants consenting to take part (Table 1). Throughout the nine-month data collection period, homeless people were invited to take part and those consenting to participate were included.

### 2.2. Oral Health

1. Obvious Decay Experience

Obvious decay experience was assessed using the DMFT index in accordance with the National Dental Inspection Programme Basic Inspection procedures and the British Association for the Study of Community Dentistry guidelines, both of which state that this is “in accordance… with international epidemiological conventions, thus allowing for comparisons to be made with other countries in Europe and beyond.” [13] (p. 5). The dental status was recorded as obvious decay experience (D_3cv_MFT), which recognised decay at the dentinal level (D_3_), with visual cavitation (D_3cv_) present. Obvious decay experience is the total D_3cv_MFT, which is a sum of the decayed into dentine with cavitation (D_3cv_), missing (M), and filled (F) teeth. 

2. Assessment of Oral Hygiene Status: Plaque

Plaque scores were assessed using the Simplified Oral Hygiene Index (OHI-S) scale of debris present [14,15,16]. Plaque scores were assessed on six teeth, if present, with scores being given as follows: “0 = no debris or stain present; 1 = soft debris covering not more than 1/3 of the tooth surface, or presence of extrinsic stains without other debris, regardless of surface area covered; 2 = soft debris covering more than 1/3, but not more than two thirds, or exposed tooth surface; 3 = soft debris covering more than two thirds of exposed tooth surface” [12] (p. 35). 

3. Oral Mucosa

An examination of the oral mucosa included the lips, buccal mucosa, tongue, floor of the mouth, palate and fauces. A score was allocated if a lesion was absent (0), lesion present and monitor (1), or requiring immediate referral (2). 

An oral health survey collection form captured all of the information regarding the participants’ obvious decay experience, plaque present, the number of standing teeth, and the incidence of oral mucosal lesions. The oral health examinations were conducted following completion of the questionnaire. The equipment used was a Daray light, disposable mirror, tweezers, and a WHO periodontal probe [17,18]. Other items, such as cotton wool pellets and rolls, were used where it was necessary to remove debris to visualize the oral structures. 

The full examination was conducted under standardized conditions observing normal infection control protocols [19]. To ensure standardized data collection, prior to the survey commencement, the 11 dentists and 12 dental health professionals who were involved in the oral examination attended a training day where they were standardized using National Dental Inspection Programme (NDIP) training materials [20]. One month prior to this training day, the practitioners had been calibrated in accordance with NDIP. 

### 2.3. The Questionnaire

The questionnaire consisted of four parts: 

1. Demographic profile.

The questionnaire asked about the participants’ age, gender, current and past living status, family status, previous occupation, and reason(s) for homelessness. 

2. Medical history and health behaviors

This section examined the participants’ medical history, including prescribed medication and health behaviors, such as alcohol, tobacco, and drug use. 

3. Psycho-social status

Dental anxiety was assessed using the Modified Dental Anxiety Scale (MDAS) [21]. The MDAS consists of five questions assessing dental anxiety in relation to: waiting for dental treatment, drilling, scale and polish, and local anesthesia. Respondents rate their dental anxiety on a five-point scale, which ranges from not anxious (1) to extremely anxious (5). Possible scores range from 5 to 25, with scores over 19 indicating dental phobia. The normative value for a general practice patient population is 10.39 and the normative value for a UK general public population is 11.60 [22].

Oral Health Related-Quality of Life was assessed using the Oral Health Impact Profile (OHIP-14) [23]. This 14-item inventory was based on a hierarchy of impacts arising from oral disease, ranging in severity, and includes functional limitation (e.g., pronouncing words), physical pain (e.g., painful aching mouth), psychological discomfort (e.g., feeling self-conscious), physical disability (e.g., interrupted meals), psychological disability (e.g., feeling embarrassed), social disability (e.g., irritable with others), and handicap (e.g., life less satisfying). Respondents were asked how frequently they had experienced each of the 14 impacts, on a five-point Likert scale, with scores ranging from 0 (never) to 4 (very often). 

Depression was measured using the valid and reliable Center for Epidemiological Studies Depression Scale (CES-D) [24]. The CES-D is a self-reported scale consisting of twenty items reflecting dimensions of depression, such as depressed mood, feelings of hopelessness, and interactions with others. The questions are answered on a four-point Likert scale and the respondents are asked to rate their experience of each item in the previous week, the responses ranged from rarely or none of the time (scoring 0) to most or all of the time (scoring 3). Total scores range from 0 to 60, with scores of 16 or over indicating depressed mood.

4. Previous dental experiences and dental health attitudes

The final part of the questionnaire inquired about the time and reason for the respondents’ most recent dental attendance, as well as previous dental treatment experiences (e.g., fillings and extractions). Opinions about going to the dentist were also assessed, using nine attitudinal measures from the Adult Dental Health Survey [25], where responses were made on a four-point Likert scale, ranging from ‘definitely feel like that’ to ‘don’t feel like that’. 

### 2.4. Administration of the Questionnaire

All dental health professionals and health practitioners who were involved in the administration of the questionnaire were provided with training tailored towards improving the understanding of the questionnaire prior to deployment, and how to engage with and assist participants with completion of the questionnaire items without influencing their responses. The participants were asked to complete the questionnaire prior to the oral examination. Many participants required help with completing the questionnaire due to poor eyesight and/or poor literacy skills. 

### 2.5. Ethical Considerations

The National Research Ethics Service was contacted concerning the requirement for ethical approval. The Integrated Research Application System (IRAS) responded to state that ethical approval from an NRES was not required. This information was provided to each of the NHS Boards who obtained the relevant NHS Research and Development Management Approval. Ethical approval was obtained from the University of Dundee Research Ethics Committee (UREC 9005). Information sheets detailing each aspect of the survey, together with written consent forms, were provided to each participant. Homeless people were given an information sheet and a consent form. All participants were required to provide informed and written consent prior to taking part. 

### 2.6. Statistical Analysis

The data was coded and entered onto a computer using SPSS version 19. Frequency distributions, *t*-tests, and regression analysis were performed on the data. 

## 3. Results

### 3.1. Sample

A convenience sample of 853 people took part in the survey. There were 598 (70%) complete data sets, as some sections were not answered by all participants: for example, 45% did not give an occupation, 10% did not answer questions about their living status, and 36% did not give a reason for their homelessness. Eighty-five percent (726) of participants had an oral examination. The results shown below report on the complete data on each variable. 

### 3.2. Oral Health Status

#### 3.2.1. Obvious Decay Experience

The mean D_3cv_MFT was 16.9 (95% CI: 16.3, 17.6). The largest component was missing teeth (8.7 [95% CI: 8.1, 9.4]), with the number of missing teeth ranging from 0 to 32. The mean number of decayed teeth into dentine with visual cavitation was 4.5 (95% CI: 4.1, 4.9), with a range of 0 to 30. The mean number of filled teeth was 3.8 (95% CI: 3.5, 4.1). The number of filled teeth ranged from 0 to 25 teeth (Table 2). Female participants had significantly fewer mean numbers of filled teeth than men (*t* = 2.22, *p* < 0.05). 

#### 3.2.2. Assessment of Oral Hygiene Status: Plaque

The total mean plaque score for the sample population was 1.08 (95% CI: 1.01, 1.15). The mean plaque score for the upper teeth was 1.06 (95% CI: 0.99, 1.13) and for the lower teeth 1.10 (95% CI: 1.04, 1.16). 

#### 3.2.3. Oral Mucosa

The oral examination assessed the six areas of the mouth and throat that are listed in the methods section. The most frequent location of a suspicious lesion was in the buccal mucosa (4%), followed by the lips (3%), palate (2%), tongue (1%), floor of the mouth (0.3%), and throat (0.2%). Overall, 61 participants (9%) had one suspicious oral mucosal lesion and six participants had two. 

#### 3.2.4. Edentulousness

Forty-six (6%) of the 726 participants who underwent the oral examination had no natural teeth. 

### 3.3. Demographic Profile

Seventy-four percent (629) of the participants were male, with ages ranging from 16 to 78. The mean age was 33.9 (95% CI: 33.1, 34.7). Age was divided into five age groups; with 207 participants being aged between 16–24 years; 194 being aged between 25–34 years; 160 being aged between 35–44 years; 160 being aged between 45–54 years; and, 51 being aged 55 years and over. Of those who answered the question on family type (805), 77% reported that they were single, with 13% having a partner and 4% and 6% being part of a one-parent family and two-parent family, respectively. 

Six hundred and ninety-four participants (81%) answered the “Living status” section, with 83 participants not responding and 76 people giving more than one answer. From those that did respond, 560 were classed as “houseless” (73%) and 46 were “roofless” (6%). 

Occupation/previous occupation was taken as an indicator of socio-economic position [26]. Of those that did provide information about their occupation, 25% worked in skilled trade occupations and 22% worked in unskilled occupations. Forty-five percent of participants did not provide details about their current or previous occupation and were assumed to be “economically inactive” [12] (p. 41).

### 3.4. Reasons for Becoming Homeless

Of the 542 participants that provided a reason for homelessness, the most frequent reason was family breakdown (22%), followed by imprisonment (11%), alcohol (9%), domestic violence (8%), drug misuse (7%), financial difficulties (6%), mental or physical ill-health (4%), relocation (3%), and unemployment (2%).

### 3.5. Medical History and Health Behaviors

Of those that completed the medical history (787), 54% reported that they were currently receiving medical treatment. Twenty-two percent reported having chest diseases; 13% reported suffering from hypertension, 7% had epilepsy, 7% had heart disease, and 3% had diabetes. Eleven percent of respondents stated that they were HIV-positive or Hepatitis C-positive (11%). 

Sixty-three percent (496) of those that completed the medical history also stated that they were taking prescribed medication, and 472 of the 496 provided the name and type of medication that they were prescribed. The most commonly mentioned prescribed medications were psychotrophic drugs (i.e., antidepressants (32%), anxiolytics (20%), and anti-psychotics (11%)) and methadone (32%). 

When asked about alcohol and tobacco consumption, 29% (240) of respondents stated that they drank alcohol “most days” and 85% (702) reported that they smoked tobacco. 

Regarding drug use, 68% of respondents reported that they had a history of street drug use. Of the 68%, 236 (29%) reported that they were currently using street drugs and of the 236, 191 stated that they were currently injecting drug users. With regard to age, significantly lower proportions of those aged 55 years and over as compared with the other lower age groups that stated that they had ever used drugs (X2[4] = 121.60, *p* < 0.001), were currently using drugs (X2[4] = 37.12, *p* < 0.001) or were injecting drug users (X2[4] = 51.34, *p* < 0.001). Equivalent proportions of male (68%) and female (66%) respondents reported to have used street drugs; currently using drugs (male: 30%; female 26%) and being injecting drug users (male 23%; female 29%).

### 3.6. Dental Anxiety Status

Of the 799 participants who completed the MDAS, the mean score for dental anxiety was 12.1 (95% CI: 11.6, 12.6). Twenty percent (170) scored over 19, which indicates that they were dentally phobic. Women as compared to men had significantly higher mean scores for dental anxiety (*t* = 5.85, *p* < 0.001). This sample was split into higher and lower dental anxiety—respondents who scored 12 or less (324) were categorized as having lower dental anxiety, while those that scored 13 or higher (475) were deemed to have high dental anxiety. The respondents with higher dental anxiety had a significantly higher mean number of filled teeth when compared to the lower dental anxiety group, whereas those with lower dental anxiety had significantly higher mean numbers of decayed teeth as compared to those with higher anxiety. There were no other significant differences (Table 3). 

### 3.7. Oral Health Related Quality of Life

**Figure 2:**
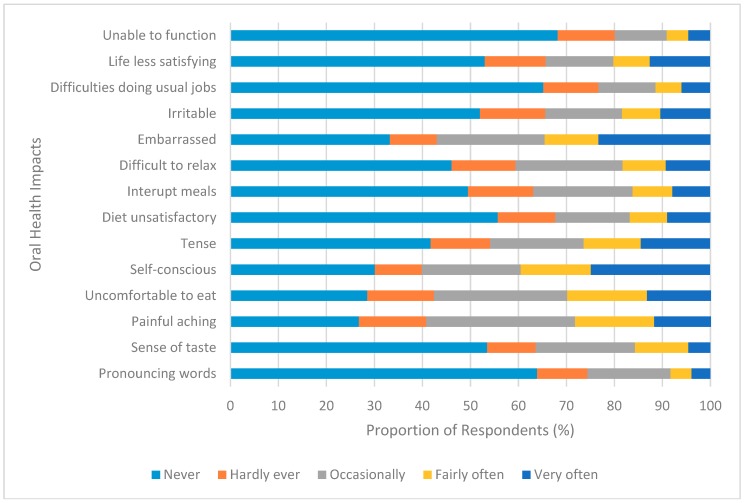
Frequency of oral health impacts.

Seven-hundred and thirty-two participants completed the OHIP-14 section of the questionnaire. The mean score for oral health impacts was 17.1 (95% CI: 16.0, 18.1). Women experienced significantly more oral health impacts when compared to men (*t* = 2.39, *p* < 0.05). The oral health impacts that were reported by participants are shown in Figure 2. Twenty-five percent (200) of participants felt self-conscious and 23% (190) felt embarrassed very often about the appearance of their mouth and teeth. The oral health impact ‘painful aching’ was experienced occasionally by 31% of the respondents; fairly often by 17%; and, very often by 12%. Twenty-seven percent reported that they occasionally felt discomfort while eating. 

The sample was divided into lower and higher oral health-related quality of life impact groups using a median split—those scoring 14 or less were categorized as experiencing lower impacts, while those scoring 15 or over experienced higher impacts. Significant differences were found between lower and higher oral health impact experiences for decayed, missing, and filled teeth, as well as overall obvious decay experience (Table 4). The mean numbers of decayed and missing teeth were significantly higher for those with higher oral health impact experience, while the mean number of filled teeth was significantly higher for the lower impact group. The mean D_3cv_MFT was significantly higher for those experiencing higher, rather than lower, oral health impacts.

### 3.8. Depression

Of the 562 participants who completed the CES-D, 58% (328) scored at least 16, which indicates that they were suffering from a depressive illness. The mean score for depression was 21.7 (95% CI: 20.5, 22.8). Women had significantly higher mean depression scores (*t* = 3.25, *p* = 0.001) when compared to men, with the mean score for women being 24.8 (95% CI: 22.6, 27.0) and for men 20.5 (95% CI: 19.2, 21.9). The sample was divided into “not depressed” (scores < 16) and “depressed” (scores > 16). Depressed participants had significantly higher mean numbers of decayed teeth and D_3cv_MFT as compared to participants who were not depressed (Table 5). Regression analysis was used to predict the effect of age, gender, and depression upon obvious decay experience. Age and depression significantly predicted obvious decay experience and explained 25% of the variance in the relationship F[2, 503] = 55.95, *p* < 0.001) (Table 6).

### 3.9. Previous Dental Experiences and Dental Health Attitudes

#### 3.9.1. Dental Attendance

Three-hundred and forty-six participants reported that they had been to the dentist in the last year, with 31% of respondents reporting that they were registered with a dentist (at the time of data collection). From those who gave a reason for their last dental visit, 68% reported that they attended due to “trouble with teeth” and 21% attended for a check-up.

#### 3.9.2. Previous Dental Treatment

The most frequently cited previous treatment experience was receiving an injection in the gum (92%), followed by fillings (89%) and extractions (81%). The least common treatment experience was bridgework, with only 12% of respondents undergoing this treatment.

#### 3.9.3. Dental Health Attitudes

When questioned about dental health attitudes, the number of respondents varied from 797 to 809. The most common attitude was “I’d like to be able to drop in at the dentist without an appointment”, with 62% of participants stating that they “definitely” felt like that. This was followed by “I’d like to know more about what the dentist is going to do and why” (37%).

## 4. Discussion

Policies from the Scottish Government over the last decade [6,7,8] have sought to improve access and support for homeless people accessing dental treatment. The 2005 Health and Homelessness Standards stated that “there are a wide range of health problems which are more prevalent amongst homeless people than the wider population… chronic diseases… infectious diseases.” [6] (p. 12). There was no mention, however, of oral health in this document. This changed with the Dental Action Plan [7], and the importance of oral health status was reinforced by the National Oral Health Improvement Strategy for Priority Groups, which made the oral health of homeless people a priority [8]. Therefore, to inform policy and improve accessible services, there was a need to conduct a survey to assess the oral health status and psychosocial needs of people that were affected by homelessness in Scotland.

The 853 homeless people who took part in this needs assessment reflected the profile of similar homeless populations elsewhere, as well as the composition of the Scottish homeless population, particularly in terms of age and gender distribution, with the majority of participants being male, with a mean age of 33.9 [1,27]. The majority of participants were “houseless”, instead of “roofless”, meaning that they were currently living in a hostel, temporary accommodation, or similar, and were not sleeping rough. A wide range of reasons were given for how the participants had originally become homeless. The most common reason given was family breakdown, which was also found to be a frequent reason for homelessness in North and West Belfast [27], along with substance misuse (alcohol and drug use).

The prevalence of smoking in this sample of participants was high, with 85% reporting that they smoked tobacco. This high percentage is surprising when it is contrasted with the comparatively low 23% of adults in Scotland that indicated they were smokers in the 2013 Scottish Household Survey [28]. Regarding alcohol consumption, the participants in this sample drank more than the general Scottish population: 12% of adults reported in the 2012 Scottish Health Survey that they drank more than five days in a week, as compared to the 29% of this sample who reported drinking most days [29]. A high smoking rate, coupled with regular excessive alcohol consumption places this population at a high risk of developing oral cancer [6,29]. In this sample, 61 participants were found to have suspicious oral mucosal lesions. Five of these required referral to secondary services.

Similarly, the high number of participants prescribed anti-depressants and methadone is not reflected in the general population. Reports from Information Services Division (ISD) Scotland show that approximately 11.3% of the Scottish population were prescribed some form of anti-depressant in 2010/11, while 122 people per 1000 population were prescribed methadone [30,31].

High levels of obvious decay experience, as well as the prevalence of edentulousness, indicates that homeless people in Scotland were not accessing or receiving the necessary level of treatment. The obvious decay experience of the population in this sample is poorer than that of the Scottish population as a whole, with a higher average number of missing and decayed teeth, and lower numbers of filled teeth [25]. However, the Scotland Health Survey (2012 edition) found that in 2012 10% of adults had no natural teeth, but in this sample population, only 6% of participants were edentulous [32].

The homeless population in this sample were found to have high levels of dental anxiety: 20% scored over 19 on the MDAS and were therefore classed as having high dental anxiety, or dental phobia. The proportion of the general UK population scoring above this cut-off is 11% [22]. It is possible that the dental anxiety in this population had developed due to negative past experiences of dental treatment, as those with high dental anxiety also had significantly more filled teeth as compared to those with low dental anxiety. This theory is strengthened by the finding that the low dental anxiety group had significantly more decayed teeth, indicating a poor history of dental attendance and therefore limited opportunity to have a negative dental experience—indeed, only one-third of participants were registered with a dentist at the time the questionnaire was administered.

Higher prevalence of obvious decay experience has clear implications for oral health-related quality of life, as decayed or decaying teeth can cause discomfort or pain, which in turn can have serious impacts on day-to-day functioning. Indeed, significant differences were found between high and low oral health impacts and D_3cv_MFT, with higher incidences of missing and decayed teeth associated with higher oral health impact. In the Adult Dental Health Survey, which studied the oral health of the United Kingdom, the most common impacts were categorized as physical pain, psychological discomfort, and psychological disability [25]. The findings from this assessment represented a similar result, with painful aching and discomfort (physical pain) being the most common impacts, followed by self-consciousness (psychological discomfort) and embarrassment (psychological disability). It is worth noting, however, that, when compared to the general population in Scotland, higher proportions of respondents in this survey experienced psychological discomfort and psychological disability regarding their teeth, mouth, and dentures [12].

Previous research has highlighted that depression among homeless people can be as high as up to four times the rate of the general population [33]. In this sample, the mean score for women was 24.8 and for men 20.5, which is considerably higher than that of the general population in the United Kingdom (14.2 for women and 13.4 for men), although, in accordance with the general UK population norms, women’s scores were higher than men’s [34]. Moreover, a significant relationship was shown between obvious decay experience with age and depression, suggesting that depression had an important influence upon oral health status. This is supported by the work of Coles et al., which showed that 19% of the depression could be explained by decayed and missing teeth in a homelessness population [35]. The implications of such findings are important, since they suggest the need for inclusion of oral health and multidisciplinary working between health, social care, and oral health services.

This assessment was affected by some limitations. First, participants were gathered from the more urban areas of Scotland, which allowed greater access to this group of participants, but perhaps did not allow for the collection of information from the more rural population, which may have its own unique barriers to dental treatment. Also, the response rate was particularly poor for some sections of the questionnaire, specifically “occupation” and “reasons for homelessness”. While participants may have left the “occupation” section blank because they were currently unemployed, participants may have left other sections blank because of the sensitive and potentially emotive nature of some of the questions.

In conclusion, the stressful and often apparent chaotic lifestyle of the homeless population has serious consequences for the general health and wellbeing of this group, and, more specifically, their oral health. When compared to the Scottish and UK general populations, the participants in this needs assessment had poorer oral and psychosocial health. Depression and dental anxiety were found to be more prevalent in this sample than in the general population. Similarly, smoking and alcohol consumption levels were higher than national averages, as were the number of people prescribed anti-depressants and methadone. 

These findings highlight that the oral health and psychosocial needs of the homeless population of Scotland are markedly different from those of the general population. As such, it is necessary to adopt a “bottom-up” approach, whereby people experiencing homelessness are encouraged to share their needs and concerns regarding oral health to help shape future oral health improvement interventions. A tailored approach that takes into account the psychosocial needs of the homeless population, not just their oral health, is therefore recommended as a method of improving the oral health and wellbeing of people affected by homelessness in Scotland. Indeed, following the needs assessment, an intervention, called Smile4life, was developed, alongside a Guide for Trainers resource, to help health and social care practitioners address the oral health needs of people experiencing homelessness [36]. The Smile4life Guide for Trainers intervention was recommended in Government strategy [8] as the approach to be taken by dental health and social care professionals to improve the oral health of people experiencing homelessness.

The provision of dental services should also be reconsidered. The findings from this study suggest that there is a reliance on emergency treatment, as indicated by the low prevalence of restored teeth. While that is perhaps appropriate for those in immediate need, there should also be a focus on providing preventive treatment alongside restorations for individuals that are able to access routine dental care. A comprehensive dental service that meets the differing needs of the homeless population should allow better access to services, which, in turn, should improve the oral health of this population group.

## Figures and Tables

**Table 1:** Details of data collection by participating National Health Services (NHS) Boards.

Board	Days/Times	Frequency	Staff	Venues
Board 1	Daytime only	1 session per week	1 dentist, 1 dental nurse, public health nurse administering questionnaire. Member of OHP Team to give opportunistic advice	Mainly hostels (may take place in drop-in center occasionally)
Board 2	Daytime only	1 session per week	1 dentist and 1 dental nurse	Hostels and the Salvation Army Drop-in Centre
Board 3	Daytime and occasional evenings	1 session per week	1 dentist and 1 dental nurse	Dental Clinic for Homeless People, Homeless Health Centre, indoor soup kitchen
Board 4	Daytime only	1 session per week	1 dentist, 1 dental nurse and an oral health coordinator	Hostels, residential units, day center, women’s refuge, homeless van, plus the homeless service
Board 5	Wednesdays 6–9 pm	Once a week (visits to 2 establishments per night in one area)	Team of 3: dentist, dental nurse and administrator. Survey team consists of 4 dentists, 4 dental nurses and 1 senior HPO, working on a rota	Hostels and soup kitchens
Board 6	Daytime and occasional evenings	2 sessions per week	2 dentists and 2 dental nurses	Homeless Clinic, day centers, hostels, night shelter
Board 7	Daytime only	1 session per week	1 dentist, 1 dental nurse, 1 hygienist and/or public health nurse from homelessness health team	Hostels, day rooms

**Table 2:** Dental health status by age group.

Dental Health Status	Age Group (*n*)	Mean (95% CI)
Decay into dentine, cavitated and visual (D_3cv_)	16–24 (207)	4.05 (3.34, 4.77)
	25–34 (194)	6.24 (5.37, 7.11)
	35–44 (160)	4.14 (3.48, 4.79)
	45–54 (96)	3.16 (2.34, 3.97)
	55+ (51)	2.75 (1.47, 4.02)
Missing teeth	16–24 (207)	2.90 (2.36, 3.44)
	25–34 (194)	7.97 (6.89, 9.06)
	35–44 (160)	11.86 (10.42, 13.31)
	45–54 (96)	13.40 (11.52, 15.27)
	55+ (51)	16.55 (13.30, 19.80)
Filled teeth	16–24 (207)	3.09 (2.62, 3.56)
	25–34 (194)	3.60 (3.08, 4.11)
	35–44 (160)	4.02 (3.40, 4.63)
	45–54 (96)	5.07 (4.12, 6.02)
	55+ (51)	4.02 (2.64, 5.40)
Obvious decay experience (D_3cv_MFT)	16–24 (207)	9.94 (8.92, 10.97)
	25–34 (194)	17.64 (16.53, 18.75)
	35–44 (160)	20.01 (18.73, 21.30)
	45–54 (96)	21.61 (20.18, 23.05)
	55+ (51)	23.31 (21.29, 25.34)
Standing teeth	16–24 (207)	26.45 (25.88, 27.02)
	25–34 (194)	22.43 (21.35, 23.50)
	35–44 (160)	18.51 (17.10, 19.91)
	45–54 (96)	17.03 (15.09, 18.97)
	55+ (51)	13.43 (10.37, 16.49)

**Table 3:** Comparison of low and high dental anxiety status with oral health status.

Oral Health Status	Lower Dental Anxiety Status (*n* = 271) Mean (95% CI)	Higher Dental Anxiety Status (*n* = 414) Mean (95% CI)	*t*	*p*
D_3cv_MFT	17.2 (16.1, 18.3)	16.6 (15.8, 17.5)	0.7	0.46
Decayed teeth	6.0 (5.4, 6.8)	3.5 (3.1, 3.9)	5.9	<0.05
Missing teeth	8.0 (7.1, 9.0)	9.0 (8.1, 9.9)	−1.4	0.17
Filled teeth	3.2 (2.8, 3.7)	4.1 (3.7, 4.5)	−2.9	<0.05

**Table 4:** Comparison of low and high oral impact experience with obvious decay experience.

Oral Health Status	Low Oral Health Impact Experience (*n* = 338) Mean (95% CI)	High Oral Health Impact Experience (*n* = 298) Mean (95% CI)	*t*	*p*
D_3cv_MFT	14.6 (13.7, 15.7)	19.2 (18.2, 20.0)	−6.5	<0.05
Decayed teeth	2.8 (2.4, 3.2)	6.4 (5.7, 7.1)	−8.8	<0.05
Missing teeth	7.8 (6.8, 8.8)	9.5 (8.6, 10.5)	−2.4	<0.05
Filled teeth	4.0 (3.6, 4.5)	3.3 (2.9, 3.8)	2.2	<0.05

**Table 5:** Comparison of obvious decay experience with depression.

Oral Health Status	Not Depressed (*n* = 222) Mean (95% CI)	Depressed (*n* = 297) Mean (95% CI)	*t*	*p*
D_3cv_MFT	14.0 (12.8, 15.3)	17.4 (16.5, 18.3)	−4.3	<0.05
Decayed teeth	3.8 (3.1, 4.4)	5.5 (4.8, 6.2)	−3.7	<0.05
Missing teeth	7.0 (5.9, 8.2)	8.2 (7.2, 9.1)	−1.5	0.13
Filled teeth	3.3 (2.8, 3.8)	3.7 (3.3, 4.2)	−1.3	0.19

**Table 6:** The effect of age, gender and depression as predictors of obvious decay experience.

Independent Variables	B	SE	*t*	*p*
Gender	−0.12	0.79	−0.14	0.89
Age	3.29	0.28	11.84	<0.001
Depression	0.09	0.02	3.73	<0.001

F[2, 503] = 55.95, *p* < 0.001: R^2^ = 0.25.
